# Effects of Admixtures on Energy Consumption in the Process of Ready-Mixed Concrete Mixing

**DOI:** 10.3390/ma15124143

**Published:** 2022-06-10

**Authors:** Veerabadrasamy Arularasi, Thamilselvi Pachiappan, Siva Avudaiappan, Sudharshan Naidu Raman, Pablo Guindos, Mugahed Amran, Roman Fediuk, Nikolai Ivanovich Vatin

**Affiliations:** 1 Department of Civil Engineering, College of Engineering Guindy, Anna University, Chennai 600025, India; drselvi08@gmail.com; 2 Departamento de Ingeniería Civil, Universidad de Concepción, Concepción 4070386, Chile; savudaiappan@udec.cl; 3 Centro Nacional de Excelencia para la Industria de la Madera (CENAMAD), Pontificia Universidad Católica de Chile, Av. Vicuña Mackenna 4860, Santiago 8331150, Chile; pguindos@uc.cl; 4 School of Engineering, Monash University Malaysia, Jalan Lagoon Selatan, Bandar Sunway 47500, Malaysia; sudharshan.raman@monash.edu; 5 Department of Civil Engineering, College of Engineering, Prince Sattam Bin Abdulaziz University, Alkharj 16273, Saudi Arabia; 6 Department of Civil Engineering, Faculty of Engineering and IT, Amran University, Amran 9677, Yemen; 7 Polytechnic Institute, Far Eastern Federal University, 690922 Vladivostok, Russia; fedyuk.rs@dvfu.ru; 8 Peter the Great St. Petersburg Polytechnic University, 195251 St. Petersburg, Russia; vatin@mail.ru

**Keywords:** ready-mixed concrete, energy consumption, mixing, ground granulated blast-furnace slag (GGBS), fly ash, self-compacting concrete

## Abstract

The production and utilization of concrete and concrete-based products have drastically increased with the surge of construction activities over the last decade, especially in countries such as China and India. Consequently, this has resulted in a corresponding increase in the energy used for the production of ready-mixed concrete. One approach to reduce the cost of concrete manufacturing is to reduce the energy required for the manufacturing process. The main hypothesis of this study is that the power required for mixing the concrete can be reduced through the use of mineral admixtures in the mix design. Optimization of energy consumption during mixing using admixtures in concrete manufacturing is the predominant focus of this article. To achieve this objective, power consumption data were measured and analyzed throughout the concrete mixing process. The power consumption curve is the only source to distinguish the behavior of the different materials used in the concrete in a closed chamber. In the current research, fly ash and ground granulated blast-furnace slag (GGBS) were used as mineral admixtures to produce ready-mixed concrete. The experimental study focused on the influence of GGBS and fly ash on power consumption during concrete mixing. The results indicated that the use of a higher content of GGBS is more beneficial in comparison to the use of fly ash in the mix due to the lower mixing time required to achieve homogeneity in the mixing process. It was found that the amount of energy required for mixing is directly related to the mixing time for the mix to achieve homogeneity.

## 1. Introduction

Energy consumption is a major issue that is faced globally in the current scenario. In all manufacturing industries, identifying and controlling the processes that consume more energy is a key element in optimizing energy consumption, which will save energy and increase cost-efficiency. Before applying all cost-saving methods to a single factory, all cost-saving methods should be carefully analyzed, because all manufacturing processes require different potential energy, resulting in different energy consumption and material savings [1,2,3]. Through the use of efficient materials and effective energy improvement technologies, the production cost of concrete can be reduced, thereby making the concrete manufacturing industry more successful [4]. An excellent energy-saving plan will reduce annual energy costs by 3 to 10% [1]. The use of energy-saving methods in the manufacturing industry can also reduce waste generated in the production process [3,5,6]. Energy guidelines are provided to identify technical deficiencies that consume more energy. These guidelines provide cost-effective practices that reduce the energy consumption of any product in the manufacturing process [7]. In addition, the task of all concrete manufacturers is to reduce energy costs without compromising product quality [8,9].

Cazacliu [10] explained the new method of concrete mixing and related energy consumption. This method explains the changes in the concrete mixture during the successive stages of concrete mixing. According to him, when concrete mixing is conducted in a closed chamber, power consumption is the only tool to identify the transition phase of the concrete mixture. The author has pointed out that the power consumption value has not been compared with the flow characteristics. Three mixer measurement methods were developed: mixing power, Orbiter (rotating microwave sensor), and Viscoprobe TM (the measurement of resistance on a spherical probe passing through the mixture) [11]. The research was based on on the relationship between the evolution of the microstructure of concrete components and the mixing time. Juez et al. [12] applied image analysis technology to monitor the mixing of concrete and observe the texture of the concrete. At the same time, the power consumption during the concrete mixing process was also recorded and correlated with the texture of the concrete with the captured images [13,14,15,16]. Chopin et al. [17] explained the increase in the homogenization time of superplasticizer concrete and its impact on concrete production. The mixing time for the SCC and HPC is increased due to the homogeneity factor. This article discusses the ability to control uniformity through power consumption measurements. Daumann et al. reported concrete mixing in single-shaft and double-shaft mixers on a laboratory scale [18,19]. The author explains the energy required for the homogenization of concrete in the mixer that can be used for different applications, including 3D-printing [20,21]. Certain characteristics of concrete mixtures will change in relation to one other, such as w/c ratio, mixing time, flow characteristics, aggregate particle size distribution curve, and compressive strength [18]. The application of energy to achieve the homogenization of different mixtures is variable. Only when all the mixtures are of the same homogeneity can the energy required for different concretes be compared. The author uses imaging measurements to determine the homogeneity of the concrete mixture. It can be seen from the above references that the influence of admixtures on concrete mixing and power consumption has not been explored. With this in mind, this article will focus on the impact of admixtures on power consumption during concrete mixing, thereby reducing energy consumption in concrete production in ready-mixed concrete plants. The workability test is also conducted for comparison with the power consumption curve. The power consumption during the mixing process also depends on the flow of the concrete; that is, the rheology of the concrete [22]. Tattersall [23] stated that increasing the water content is the final decision of workers to improve the workability of concrete. To date, the relationship between the power consumption curve and the workability of the concrete has not been concentrated on. From the above literature, it is observed that the power consumption required for the mixing of the concrete has not been studied for the ready-mix concrete. The impact of the admixtures, slump value, and water/powder ratio (w/p) on power consumption during mixing has also not been explored.

### 1.1. Importance of Mixing

Mixing is the most important stage in concrete manufacturing and is one that consumes the most energy. A ready-mixed concrete factory produces different types of concrete. Mixing plays a vital role in distinguishing different types of concrete. Nowadays, the manufacturing of SCC and HPC has increased due to emerging high-rise buildings and large structures. The power consumption curve obtained during the mixing of concrete gives a better understanding of the microstructural evolution in the concrete mixture [10]. Mixing consists of the evolution of a wet granular state to a granular microstructure suspension [11]. The mixing process is a key element in achieving good quality concrete [18,24,25]. In the current scenario, adequate mixing is not carried out by the concrete manufacturer. There is a lack of knowledge concerning the new materials used in concrete manufacturing. The increase in HPC and SCC productivity provides an opportunity to use new materials in the concrete production industry [12,26,27]. Concrete manufacturers should pay attention to the characteristics and performance of new materials and use appropriate mixing times to obtain high-quality concrete. A longer mixing time is applied in order to properly homogenize the concrete mixture, which affects the mixing efficiency. The elimination of inhomogeneity in the concrete mixture is a result of the mixing efficiency, and thus the mixing of concrete is the important phase for obtaining a quality outcome. Improper mixing leads to weak concrete and structural failure, and it has been common in the construction industry since HPC and SCC came into existence [28,29]. The production of HPC and SCC has given a higher priority to introducing new material into manufacturing concrete; hence, increasing the mixing time should be avoided in order to ensure the quality of the concrete. In the manufacturing of HPC and SCC, concrete manufactures are asked to respect the mixing time of concrete [30,31].

### 1.2. Mixing Mechanism

Mixing is an important operation that eliminates the inhomogeneity in a mixture. ASTM 305 is commonly used all around the world for mixing procedures [32]. Two important factors that can occur during the mixing operation are intensive mixing and extensive mixing [33]. Intensive mixing reduces agglomeration of the particles held by surface tension. The agglomeration can be reduced when the strength of the inter-particle bond is subject to higher hydrodynamic stresses [34]. During intensive mixing, fine powder is converted into a viscous fluid. In extensive mixing, deformation of the fluid takes place, which increases the interface area between the particles, and hence the inhomogeneity is reduced. Shear history is important for all fresh concrete because the binder-rich SCC exhibits thixotropic and structural breakdown characteristics. In the mixing of fresh concrete, the mixing energy is closely related to the shear rate [35].

### 1.3. Production and Energy Consumption of Ready-Mixed Concrete

In high-speed construction, concrete production requires more attention because there is no optimal time for concrete mixing [36]. In the past 20 years, ready-mixed concrete plants have produced a large quantity of SCC and HPC. The problems encountered in concrete mixing are mainly concentrated in ready-mixed concrete plants [37]. Measuring power consumption during mixing is important because more than 200 m^3^ of concrete can be manufactured per day in one ready-mixed concrete plant. In the future, this may increase due to the development of urbanization. In the manufacture of ready-mixed concrete, ingredients are added at specific intervals. Cement, fine aggregate, coarse aggregate, fly ash, GGBS, and water are added one by one into the mixing chamber. As the ingredients are loaded into the mixing chamber, power consumption will increase. The maximum power consumption is obtained when all components are loaded. The first peak is obtained when the mixture of dry ingredients reaches homogeneity in the dry state. After adding water, when a homogeneous mixture is obtained in a wet state, the second peak is reached [10,11]. After the homogenization is obtained, the power consumption is gradually reduced, and then the concrete is discharged.

### 1.4. The Relation between Power Consumption and the Mixing of Concrete

Power consumption is an important factor in determining the different stages of concrete mixing in the mixing chamber [10]. The power consumption curve is the only tool to determine whether homogeneity is achieved. The homogeneity of concrete varies from one mixture to another [21]. This leads to differences in concrete energy consumption. The power consumption during mixing is divided into different stages. The first stage starts from an empty condition and proceeds to the loading condition. The power consumption will gradually increase during the subsequent loading. In the second stage, the ingredients will mix thoroughly in a dry state, followed by the addition of water, where the moisture is absorbed by the powder particles [10]. At first, the power consumption does not increase because it does not form any liquid bridge. In the third stage, because a liquid bridge is formed between the powder particles, the saturation rate increases, and the gradual mixing will cause a sudden increase in power consumption. Further mixing disintegrates the granular particles to form a paste. At this stage, power consumption will reach its peak. In the fourth stage, homogeneity of the mixture can be achieved by continuously mixing the ingredients, and the concrete can reach full saturation. The starting point of the fourth stage will show higher power consumption, which will gradually decrease once a homogenous mixture is obtained [21,22,23,24].

The gaps identified from the above literatures are as follows: The power consumption required for the mixing of the concrete has not been studied for the ready-mix concrete. The impact of the admixtures, workability, and density of concrete on power consumption during mixing has not been explored.

## 2. Materials and Methods

**Figure 1:**
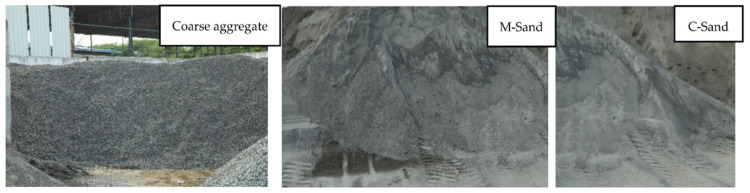
Storage of coarse aggregate and fine aggregate in the RMC plant.

**Figure 2:**
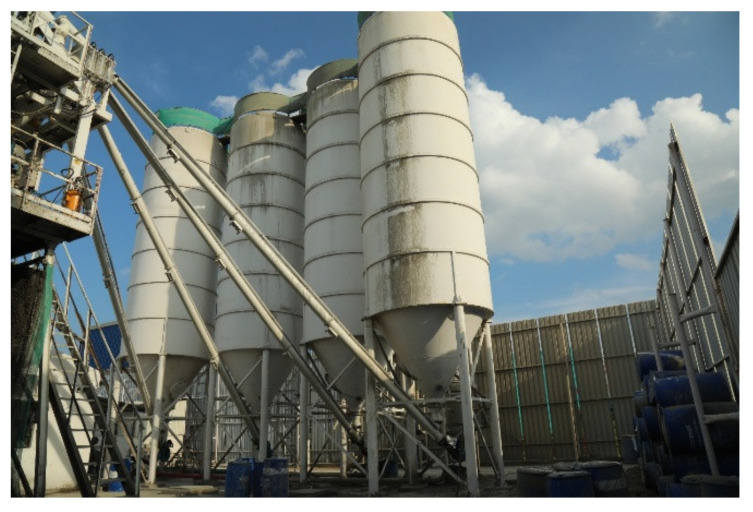
Storage of cement, fly ash, and GGBS in silos.

In this experimental investigation, energy consumption was measured during the mixing of concrete manufactured in a ready-mixed concrete plant. The mixing motor was connected to the power meter, where the voltage and current consumed during mixing were recorded. The power was calculated by the formula P = V × I. In the ready-mixed concrete plant, 1 m^3^ of concrete was manufactured for each batch. The total quantity of concrete required for one truck may vary from 6 to 8 m^3^, and it takes approximately 1 min to mix 1 m^3^ of concrete in the mixing unit. Three grades of concrete, M25, M30, and M40 were studied. From each grade, four to five mixes were produced by replacing fly ash and GGBS in different percentage, and its energy consumption during mixing was measured and the energy curve plotted. The raw materials used were cement, fine aggregate, coarse aggregate, fly ash, GGBS, and water. All raw materials were stored in different storage locations in the RMC plant, as shown in Figure 1 and Figure 2, and when the mixing operation began, they were transported to the mixing chamber by a conveyor. Two types of high-efficiency water-reducing agents were used: PCE-based (Polycarboxylic ethers), a medium-range water-reducing agent, and SNF-based (Sulfonated naphthalene formaldehyde), a high-range water-reducing agent [37,38,39,40].

### 2.1. Composition of the Concrete Ingredients

Grade 53 ordinary Portland cement meeting IS 269-2015 (Indian Standard) was used to produce concrete in the RMC plant. Fly ash, conforming to IS 3812-2003, was obtained from the Ennore thermal power plant in India, and was used as a mineral admixture. Another mineral admixture, GGBS, conforming to IS 16714:2018, was also used. Table 1 lists the chemical composition of the binding material and Table 2 gives the physical properties of the binding materials.

M-sand and C-sand were used as fine aggregate and coarse aggregate of size 12 mm and 20 mm, respectively, both the aggregates conforming to IS 383-2016. They were obtained from the locally available market. Both types satisfy the requirements of the Indian standard that complies with grading zone II. Crushed granite stone was used as coarse aggregate with sizes of 12 mm and 20 mm. This was obtained from the local dealers of Chennai, and it satisfies the Indian standard requirement. Table 3 shows the physical properties of the aggregates.

The two chemical admixtures used were Polycarboxylate ether (PCE) and Sulphonate naphthalene formaldehyde (SNF), obtained from CBS Chemicals, and these were used as retarding Superplasticizers. Table 4 shows the chemical properties of the aggregates.

The mix design was calculated according to the Indian standard method by adopting mix code IS 10262-2019. The batching and mixing of the concrete were carried out according to the code IS 4925-2004. The entire manufacturing was carried out in a ready-mixed concrete plant. The Mix compositions and specifications are shown in Table 5 and Table 6.

### 2.2. Power Meter Reading

**Figure 3:**
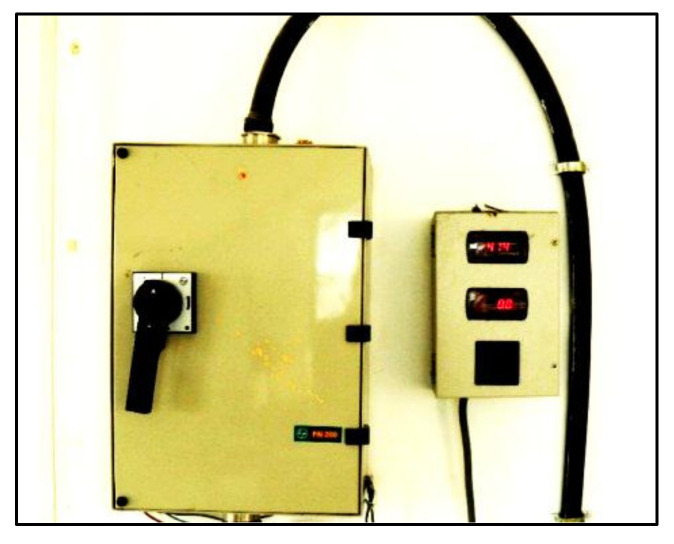
Power meter shows the reading of voltage and current during the mixing operation.

**Figure 4:**
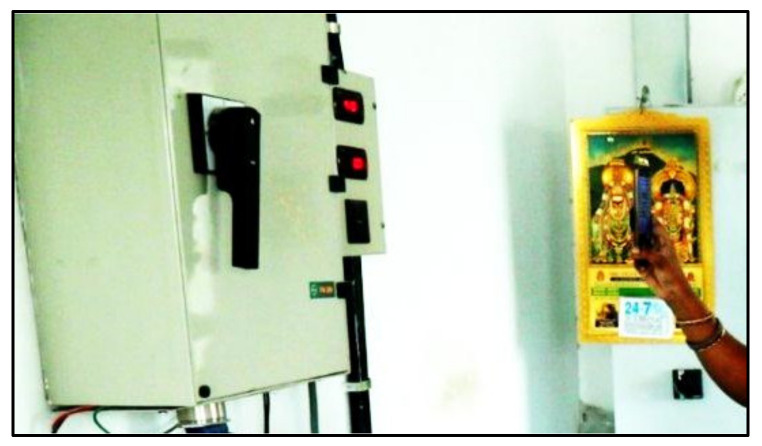
Recording the power meter reading during the mixing operation.

The entire power meter readings during the mixing process were recorded, as shown in Figure 3 and Figure 4. A 100 amp watt-meter was connected to the mixer machine. Power consumption was noted down for every second to plot the power curve for the concrete manufactured.

**Figure 5:**
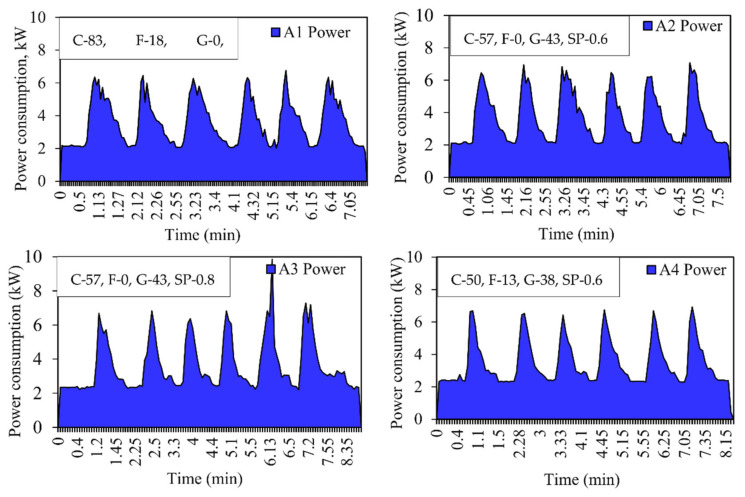
Energy consumption curve for M25 grade concrete for Mix A1, A2, A3, and A4. C—Cement, F—Fly ash, G—GGBS, SP—Superplasticizer, (expressed in %).

**Figure 6:**
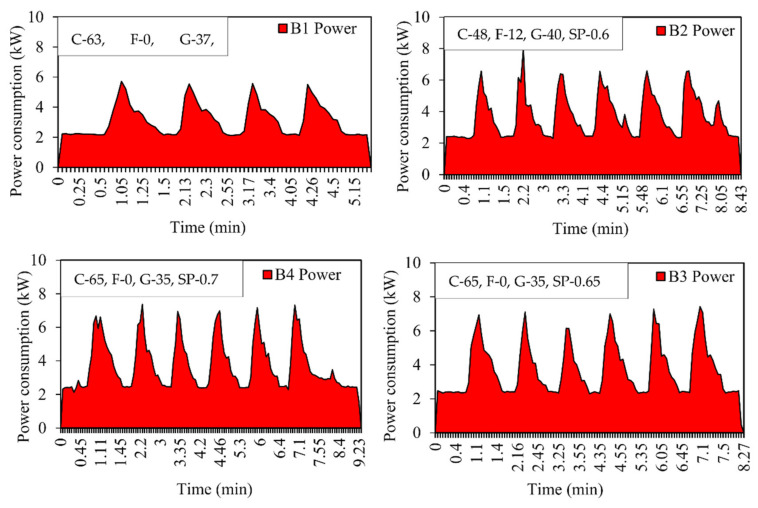

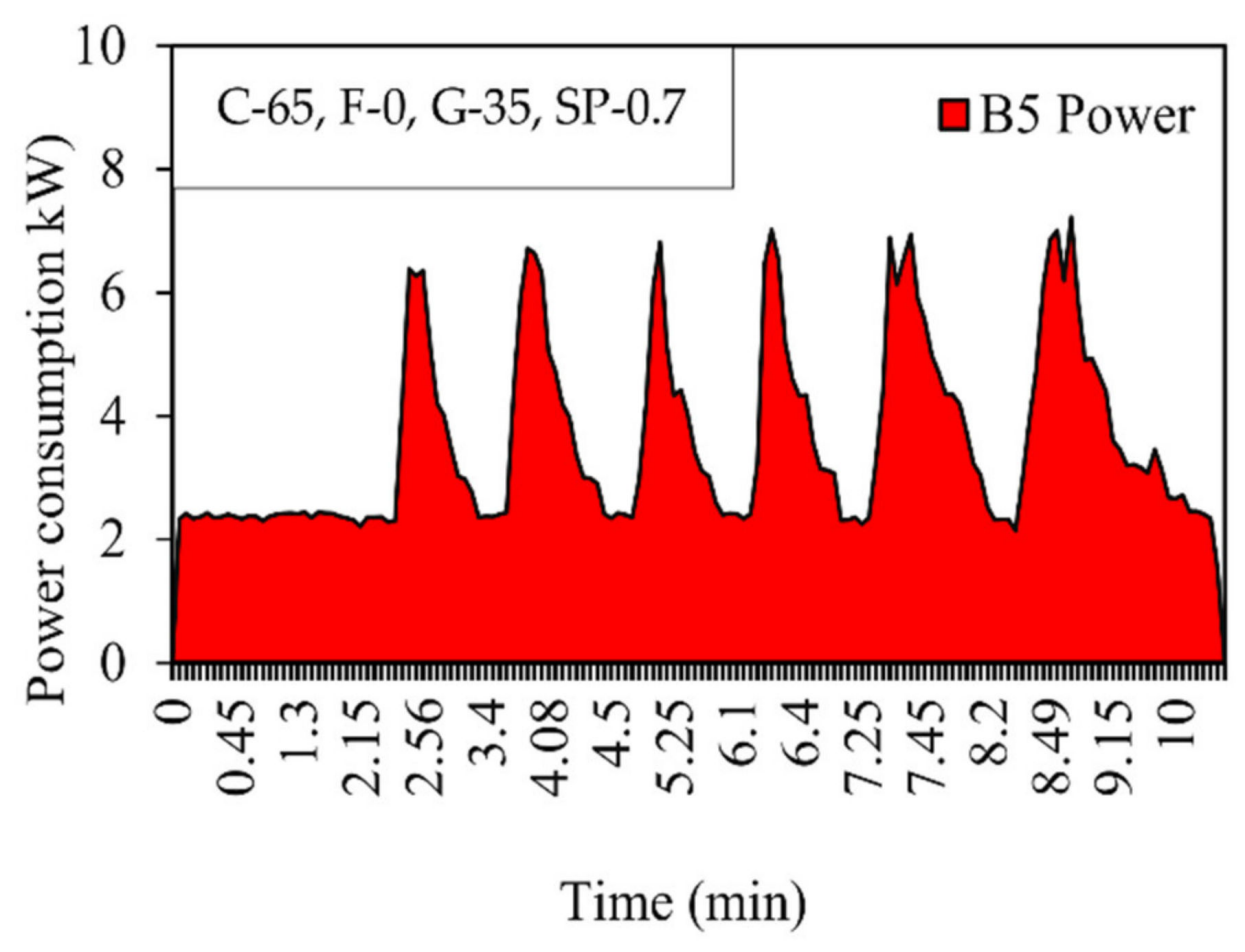
Energy consumption curve for M30 grade concrete for Mix B1, B2, B3, B4, and B5. C—Cement, F—Fly ash, G—GGBS, SP—Superplasticizer (expressed in %).

**Figure 7:**
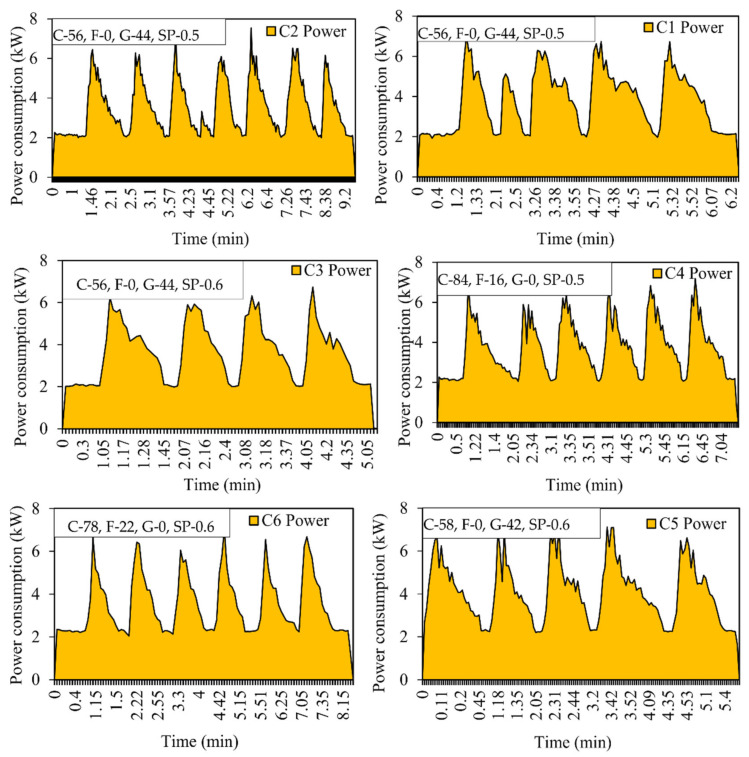
Energy consumption curve for M40 grade concrete for Mix C1, C2, C3, C4, C5, and C6. C—Cement, F—Fly ash, G—GGBS, SP—Superplasticizer (expressed in %).

The curve was plotted against time versus power consumption. Each peak represents the mixing of 1 m^3^ of concrete, i.e., one batch. The total number of peaks represents the total quantity of the concrete manufactured. It can be seen from the curve that the initial reading starts at zero, and as the material is loaded, the power consumption will increase [41]. Energy consumption is calculated by the average method based on the readings obtained during concrete mixing. Figure 5, Figure 6 and Figure 7 show the power consumption curves of M25, M30, and M40.

### 2.3. Measurement of Slump Value

**Figure 8:**
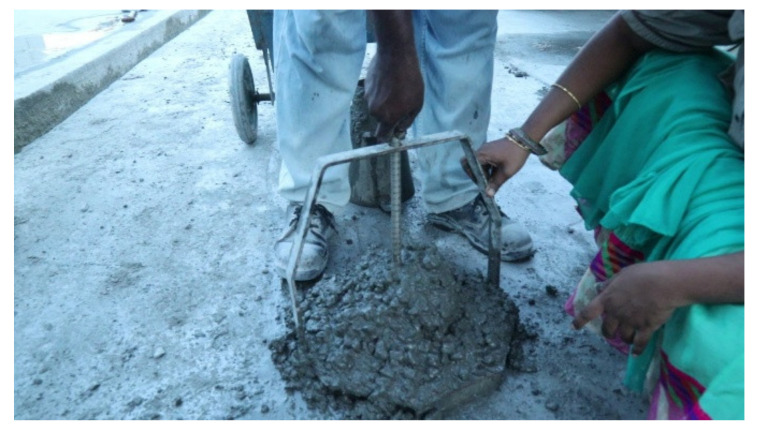
Measuring the slump value.

The slump test is used to measure the workability of concrete. Workability tests are usually performed on all batches of concrete in the ready-mixed concrete plant to check the uniformity of the different batches of concrete manufactured. The slump test will show different values for the same concrete manufactured in the morning and afternoon. The amount of a chemical admixture depends on the slump value of the first batch of concrete produced. After the concrete is discharged from the mixer, the freshly cast concrete is directly composed from the outlet of the truck mixer to perform a slump test. The entire test is carried out in the ready-mixed concrete plant. Furthermore, this research has been extended to correlate power consumption with a slump value, which has not been carried out previously for ready-mixed concrete. The measurement of the slump value is shown in Figure 8.

## 3. Results and Discussion

**Figure 9:**
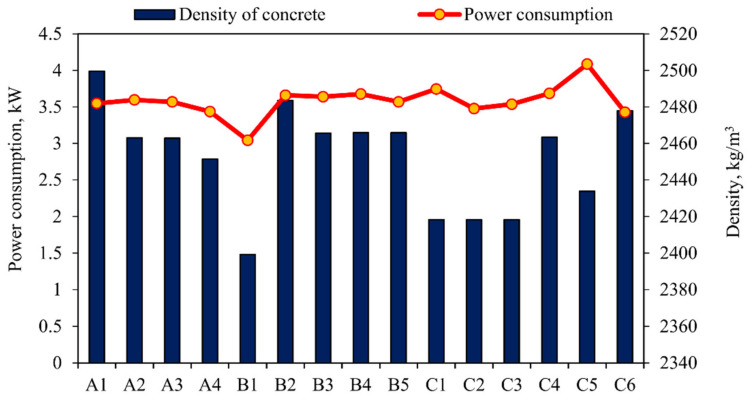
Power consumption versus density of concrete.

**Figure 10:**
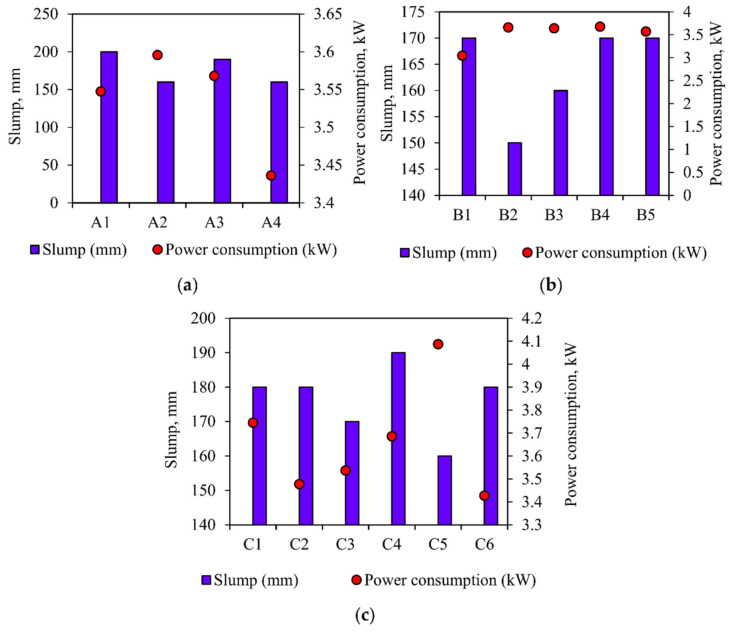
Power consumption versus slump value for (**a**) M25 grade concrete, (**b**) M30 grade concrete, (**c**) M40 grade concrete.

**Figure 11:**
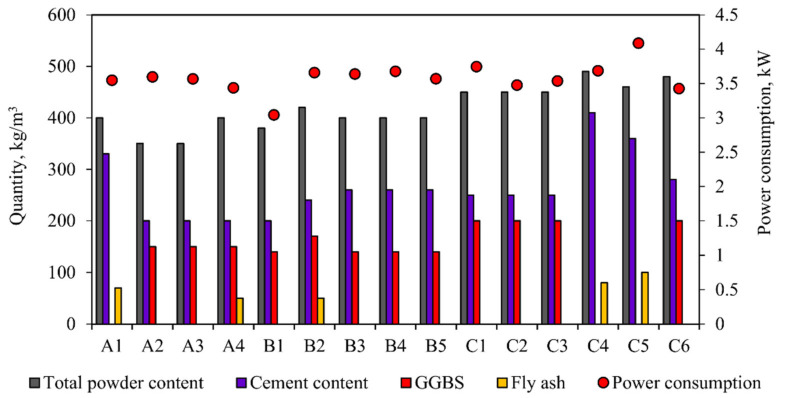
Power consumption versus total powder content (GGBS + fly ash + cement), GGBS, cement content, and fly ash.

**Figure 12:**
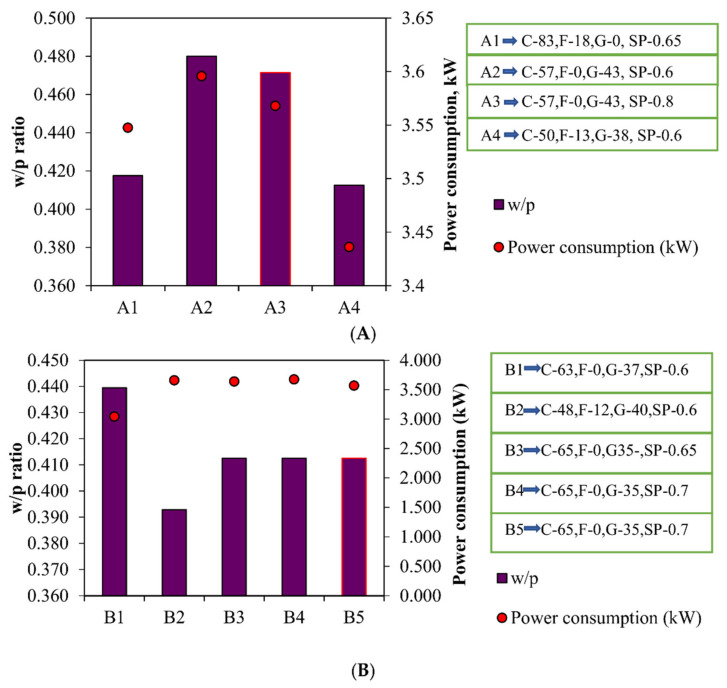

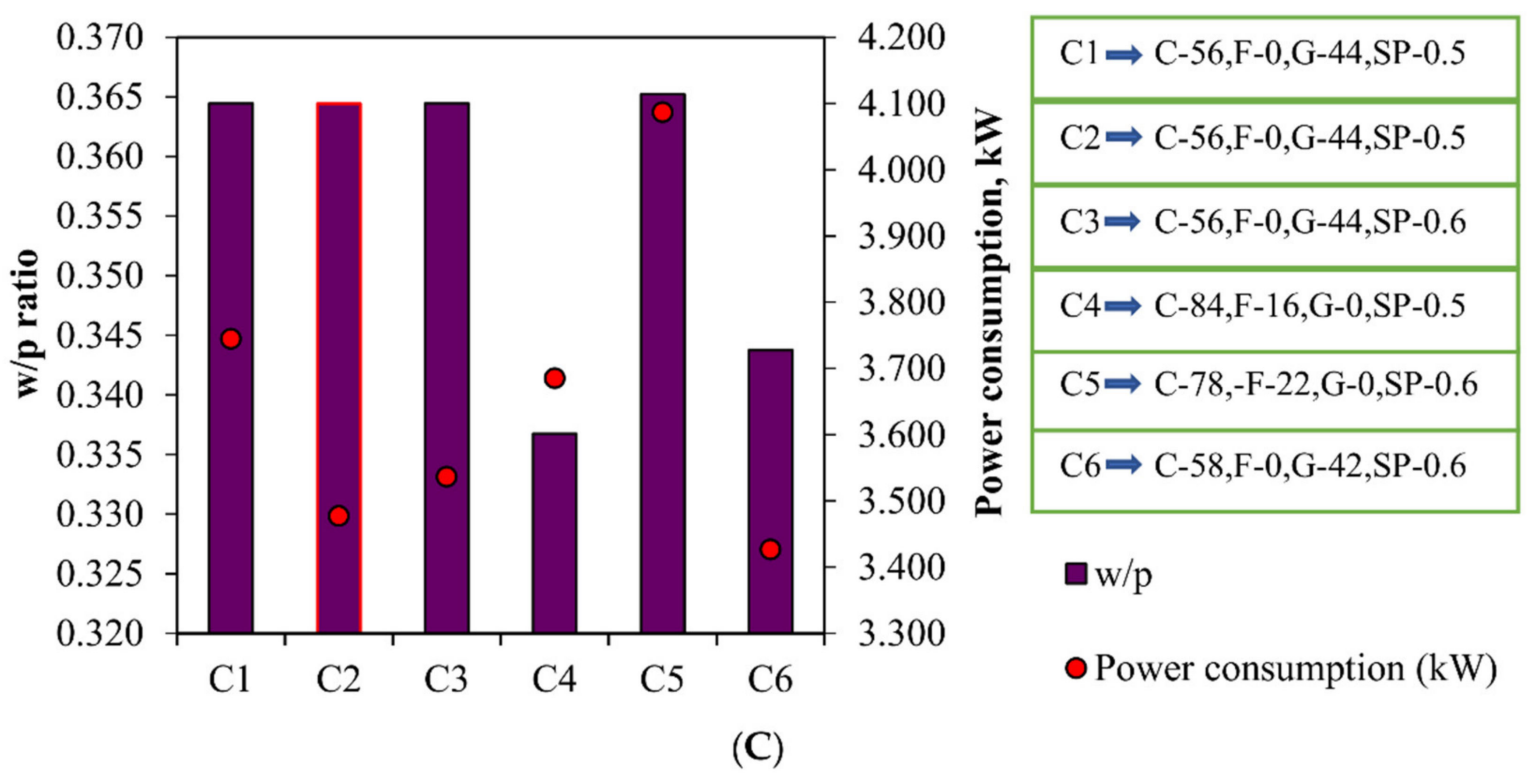
Power consumption versus w/p ratio for (**A**) M25 grade concrete, (**B**) M30 grade, (**C**) M40 grade concrete. C—Cement, F—Fly ash, G—GGBS, SP—Superplasticizer. A3, B5, and C5, 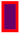
—SNF is used and for other mixes, 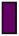
—PCE is used.

The power consumption for the various grades of concrete and the slump value for the corresponding mixes are shown in Table 7. The reason for the increase or decrease in the power consumption for the various mixes is explained in Figure 9, Figure 10, Figure 11 and Figure 12. The slump value obtained for the various mixes is compared to the power consumption to find the workability of the concrete and its impact on power consumption.

It can be seen from Figure 9 that the power consumed by the high-density concrete A1 is almost the same as that of the lower density mixtures A2 and A3. The lower density mixture A4 consumes the least power. In M30 concrete, the density of the mixture of B2, B3, and B4 does not increase or decrease much, even though the density of B2 is very high. In B1, for the minimum density concrete, power consumption is significantly reduced. In the M40 low-density concrete, C1 and C5 indicate higher energy consumption, while higher-density concrete C6 indicates the minimum energy consumption.

From the above observation, we can clearly understand that density is an important factor in power consumption, but it is not the only factor that controls power consumption. The M25- and M30-grade concrete shows similar characteristics in power consumption, i.e., lower density mix consumes minimum power and higher mix consumes maximum power. In higher-grade concrete (M40), the results show the opposite characteristics, namely, high-density concrete consumes the least power, while low-density concrete consumes the most power [42,43], though this does depends on the material used for the concrete mix [44,45,46]. In high-grade concrete, the powder content is usually higher than that of low-grade concrete, as shown in Figure 11. The dosage of the chemical mixture and water content is another important factor in increasing or reducing power consumption. The powder (cement + fly ash + GGBS) in the concrete and the water and chemical admixtures used are important factors that can increase or decrease power consumption during the concrete mixing process, as shown in Figure 10, Figure 11 and Figure 12.

In Figure 10a, it can be seen that the power consumption decreases with the increase of the slump value, which is obvious in A1, A3, and A4. Mix A2 has a lower slump value and consumes more power during mixing, while A1, A3, and A4 have a higher slump value and consume the least power. 

In Figure 10b, similar to the previous one, the mix B2 obtains the lower slump value and consumes the maximum power. In contrast, the slump of the mixtures B1, B3, B4, and B5 is higher, and the power consumed during the mixing process is minimal. Similarly, in Figure 10c, C5 reduces slump and consumes maximum power during mixing. The other mixes, C1, C2, C3, and C6, have a higher slump and consume the least power.

The mixtures C1 and C2 have similar compositions and slump values, but the power consumption of C2 is abridged. This reduction may be due to the use of SNF superplasticizer. The composition of the mixtures B4, B5, A2, and A3 are similar, but the purpose of the water-reducing agent is different. SNF is used as a chemical mixture in A3 and B5, which is further explained in Figure 12 below. Certain mixtures (such as A4, B1, and C6) are unique and show very low power consumption. This may be due to the availability of the powder content in the mixture, and therefore requires further inspection, which is explained in the comparison between the w/p ratio and the power consumption.

Figure 11 explains the effect of powder content on power consumption. In M25 concrete, the power consumed by A2 is very high, and it consists of the powder content in the combination of cement and GGBS. Mixture A4 consumes the least power and contains a higher content of cement, fly ash, and GGBS. In M30 concrete, the minimum power is consumed by B1, which is composed of cement and GGBS. Mixture B2, which is a combination of cement, fly ash, and GGBS, consumes the maximum power. In M40 concrete, the minimum power is consumed by mixture C6, which includes cement and GGBS. Mixture C5 consumes the maximum power, and is composed of the powder content of cement and fly ash.

The amalgamation of powder content for each concrete has a different effect on power consumption. In most cases, the combination of cement and fly ash consumes more power during the mixing process, while the combination of cement and GGBS consumes the least power [21,26,47,48]. When observing these three grades of concrete, it can be seen that the combination of powder content is another factor that affects power consumption. The molecular structure of the powder enhances the smooth mixing of the concrete in the fresh state [49]. Smooth mixing and rough mixing will cause differences in power consumption [50]. The powder is mixed with water and superplasticizer to form granules. These pastes will bind fine and coarse aggregates, causing wear on the surface of the mixing drum. The molecular structure of the paste is the main factor in reducing the inter-particle wear between the components and the wear on the sidewall of the mixing drum [51]. Therefore, Figure 12A–C further explains the behavior of powder and water in order to provide a clear understanding of the energy consumption of concrete during mixing.

In Figure 12A, A1 and A4 show the minimum power consumption as the w/p ratio decreases. The power consumption increases as the w/p ratio increases. When comparing these two mixtures, A1 and A4, with similar w/p ratios, it can be seen that the combination of powders is an important criterion for increasing or reducing power consumption. In A1, the combination of cement and fly ash consumes more power than A4 (composed of a combination of cement and GGBS). The composition of the mixtures A2 and A3 is similar, but the dosage and type of the water-reducing agents are different. In A4, SNF superplasticizer is used, which has the lowest power consumption. In Figure 12B, when cement and GGBS are combined, B1 consumes the lowest power and has a very high w/p ratio. The mixture B2 consumes more power for low w/p ratio with a combination of cement, fly ash, and GGBS. In a similar mix of B4 and B5, B5 has a smaller power consumption than B4.

Among C1, C2, and C3, which have similar compositions but differ in the type and dosage of superplasticizer, C2, which contains SNF as a superplasticizer, consumes minimum power during mixing. With the combination of fly ash and cement, the w/p ratio increases and mixture C5 consumes the maximum power. Of the mixtures C4 and C6, mixture C4, consisting of cement and fly ash, consumes the maximum power, while mixture C6, composed of cement and GGBS, consumes the minimum power.

The enlargement of the particle size is called granulation, and it depends on the powder used in the mixing process [49,51,52]. Spherical particles and well-compacted particles reduce the duration of the mixing operation. In the shear mixture, the formation of granules is a very sensitive phenomenon that controls the mixing operation. In high-shear mixtures, the characteristics of the powder content and the formation of granules both have an impact on the power consumption during the mixing operation. The amount of binder also controls the mixing operation and reduces power consumption. The cohesive force involved in the agglomeration also causes an impact on power consumption [53,54]. Cohesive force is caused by the liquid bridge between the solid particles and relies on the surface tension of the granulation liquid. The force required to separate the agglomerates, which are achieved by the coalescence of many primary particles, will always be higher than the force required for the nucleation of the primary particles. The addition of viscous material can reduce the separating force of the liquid bridge [54,55,56,57]. In this study, granules consisting of fly ash and GGBS are responsible for the increase or decrease in power consumption, respectively. The well-defined spherical shape of GGBS forms granules, which can decrease friction during the mixing operation and therefore decrease power consumption.

## 4. Conclusions

The change in power consumption is primarily a result of the different materials included to the concrete mixture. The mineral admixtures used in concrete have different chemical and physical properties. As a result, all materials act differently. Identifying the materials that can minimize energy consumption and using them in concrete production can reduce the energy required for the manufacturing process.

Energy optimization in all areas is the ultimate goal in the protection of our natural resources. It can be seen from the above results that the maximum use of powder content has an impact on the energy consumption, thereby achieving the homogeneity of the concrete during the mixing process. However, the following conclusions were drawn based on the major findings of this study:

– A maximum fly ash content of 22% increases the power consumption by 19.27–20.28%. Inversely, a minimum fly ash content of 16–18% decreases the power consumption by 1.3–9.83%. A GGBS content of 35–44% increases the power consumption by 3.8–20.8%.
– For the slump value, 16–18% of fly ash increases the value by 12.5–25%, and hence there is a decrease in the power consumption. A fly ash content of 22% decreases the slump by 11.8 to 15.8%, and there is a corresponding increase in the power consumption by 19.27 to 20.28%. There is an increase in the slump value of 6.7–16.8% for 35–44% GGBS content.
– It is obvious from the findings that the maximum use of fly ash will consume more energy to achieve homogeneity and increase the energy required for concrete production.
– For concrete containing fly ash, its curve profile, which is visible in the energy consumption curve, rises and falls unevenly due to the formation of granules when fly ash and GGBS are mixed with water.
– The quality of the mixing depends upon the degree of homogeneity attained at the end. The homogeneity obtained depends on several factors, such as the properties of single particles, the properties of bulk particles, operating conditions, and mix design.
– It was found that an increase or decrease in water content may also lead to an increase or decrease in power consumption. The texture of the aggregate and the mineral admixtures added to the concrete can cause internal abrasion on the surface of the mixing drum.
– Aggregate and mineral admixture wear and particle collisions are other factors leading to increased power consumption. The mixer drum has difficulty blending certain materials because hard particles prevent blending and overcome the torque, so the drum consumes excess power in order to rotate. This could be one of the key reasons for the difference in power depletion when using different mineral admixtures.

## Figures and Tables

**Table 1:** Chemical composition of the used materials (expressed in %).

Components	SiO_2_	Al_2_O_3_	CaO	MgO	Fe_2_O_3_	SO_3_	S	Cl	Na_2_O	K_2_O	TiO_2_	Mn_2_O_3_
Cement	23.6	6.2	62.3	2.5	3.1	1.9	-	0.03	-	-	-	-
Fly ash	51.5	18.8	5.7	1.9	17.1	2.5	0.5	-	0.4	0.6	0.75	0.12
GGBS	32.5	11.2	41.3	10.6	2.1	0.5	-	-	-	0.32	0.6	0.35

**Table 2:** Physical properties of the used materials.

Properties	Cement	GGBS	Fly Ash
Initial setting time (min)	30	90	80
Specific gravity	3.15	2.9	2.7
Normal consistency (%)	29	-	-
Final setting time (min)	510	550	580
Specific surface area, m^2^/kg	300	375	390

**Table 3:** Properties of aggregates.

Properties	Sand #1 (Sand-C)	Sand #2 (Sand-M)	Gravel #1 (12 mm)	Gravel #2 (20 mm)
Specific gravity	2.6	2.7	2.6	2.6
Water absorption	0.9	1.7	1	1
Fineness modulus	3.3	3.4	5.2	6.5

**Table 4:** Chemical admixture properties.

Parameter	SNF	PCE
Chemical name	Sulphonated naphthalene formaldehyde	Polycarboxylic Ether
Appearance	Reddish-brown liquid	Light-brown liquid
pH	6	6
Relative density	1.11	1.07
Chloride content	0.2%	0.1%
Type	Retarding Superplasticizer	Retarding Superplasticizer

**Table 5:** Concrete Mix Compositions.

Components		Cement	C-Sand	M-Sand	Fly Ash	GGBS	Water	Superplasticizer	
Mix	Grade			kg/m^3^				Quantity (kg)	Type
A1	M25	330	432	322	70	0	167	2.6	PCE
A2	M25	200	400	400	0	150	168	2.1	PCE
A3	M25	200	458	305	0	150	165	2.8	SNF
A4	M25	200	438	292	50	150	165	2.4	PCE
B1	M30	240	360	360	0	140	167	2.28	PCE
B2	M30	200	444	290	50	170	165	2.52	PCE
B3	M30	260	382	382	0	140	165	2.6	PCE
B4	M30	260	382	382	0	140	165	2.8	PCE
B5	M30	260	382	382	0	140	165	2.8	SNF
C1	M40	250	350	350	0	200	164	2.25	PCE
C2	M40	250	350	350	0	200	164	2.25	SNF
C3	M40	250	350	350	0	200	164	2.25	PCE
C4	M40	410	363	363	80	0	165	2.45	PCE
C5	M40	360	349	349	100	0	168	2.76	PCE
C6	M40	280	350	350	0	200	165	2.88	PCE

**Table 6:** Concrete Mix specifications.

Mix	Water/Powder	Total Quantity (m^3^)	Batch Quantity (m^3^)	Duration of Mixing (Min)
A1	0.413	6	1	7.48
A2	0.48	6	1	8.15
A3	0.471	6	1	8.25
A4	0.413	6	1	5.25
B1	0.439	4	1	8.55
B2	0.393	6	1	8.4
B3	0.413	6	1	9.23
B4	0.413	6	1	10.4
B5	0.413	6	1	8.15
C1	0.364	5	1	6.34
C2	0.364	7	1	9.53
C3	0.364	4	1	5.15
C4	0.337	6	1	7.42
C5	0.365	5	1	6.08
C6	0.344	6	1	8.27

**Table 7:** The power consumption and slump value measured for different mixes.

Mix	Power Consumption (kW)	Slump (mm)
A1	3.547	200
A2	3.595	160
A3	3.568	190
A4	3.436	160
B1	3.042	170
B2	3.659	150
B3	3.638	160
B4	3.675	170
B5	3.568	170
C1	3.744	180
C2	3.477	180
C3	3.536	170
C4	3.685	190
C5	4.086	160
C6	3.426	180

## Data Availability

Not applicable.
